# Increased recurrent falls experience in older adults with coexisting of sarcopenia and knee osteoarthritis: a cross-sectional study

**DOI:** 10.1186/s12877-021-02654-4

**Published:** 2021-12-15

**Authors:** Hirotaka Iijima, Tomoki Aoyama

**Affiliations:** 1 Department of Physical Therapy, Human Health Sciences, Graduate School of Medicine, Kyoto University, Kyoto, Japan; 2 Department of System Design Engineering, Faculty of Science and Technology, Keio University, 3-14-1 Hiyoshi, Kohoku-ku, Yokohama, 223-8522 Japan; 3 Japan Society for the Promotion of Science, Tokyo, Japan

**Keywords:** Sarcopenia, Knee osteoarthritis, Recurrent falls

## Abstract

**Background:**

Sarcopenia and knee osteoarthritis (OA) are two major risk factors for falls in older adults. The coexistence of these two conditions may exacerbate the risk of falls. This cross-sectional study aimed to test the hypothesis that older adults with coexisting sarcopenia and knee OA displayed an increased risk of falls experience.

**Methods:**

Participants recruited from an orthopedic clinic were divided into four groups according to the presence of sarcopenia and radiographic knee OA: isolated sarcopenia, isolated knee OA, sarcopenia + knee OA, and control (i.e., non-sarcopenia with non-OA) groups. We used questionnaires to assess falls experience in the prior 12 months. We performed logistic regression analyses to evaluate the relationship between the four groups and falls experience.

**Results:**

Of 291 participants (age: 60–90 years, 78.7% women) included in this study, 25 (8.6%) had sarcopenia + knee OA. Participants with sarcopenia + knee OA had 4.17 times (95% confidence interval: 0.84, 20.6) higher odds of recurrent falls (≥2 falls) than controls after adjustment for age, sex, and body mass index. The increased recurrent falls experience was not clearly confirmed in participants with isolated sarcopenia and isolated knee OA.

**Conclusions:**

People with coexisting of sarcopenia and knee OA displayed increased recurrent falls experience. This study suggests a new concept, “sarcopenic knee OA”, as a subgroup associated with higher risk of falls, which should be validated in future large cohort studies.

Trial registration.

Not applicable.

**Supplementary Information:**

The online version contains supplementary material available at 10.1186/s12877-021-02654-4.

## Background

Sarcopenia, an age-dependent loss of skeletal muscle mass [1], is a major risk factor for falls in older adults [2]. As falls are the leading cause of unintentional injury [3] and subsequent fear of falling [4], fall prevention in persons with sarcopenia is important. The importance of the fall prevention is particularly true for older adults who fall recurrently (i.e., 2 or more falls per year) given that the recurrent fallers experience greater morbidity than those who are not recurrent fallers [5–8]. A key component in the prevention of falls is the identification of specific subgroup with high risk of falling [9].

A potential coexisting condition for falls in persons with sarcopenia that was not adequately addressed in previous studies is knee osteoarthritis (OA). Knee OA, a leading cause of pain and disability, is an independent risk factor for falls in older adults [10–12]. Individuals with knee OA share the same fall risk factors with sarcopenia, including impaired balance and muscle weakness [13]. Notably, in addition to these common risk factors, knee joint instability and knee pain are potential risk factors for falls unique to individuals with knee OA [13]. Collectively, these evidences lead to a new hypothesis that the coexistence of sarcopenia and knee OA, occurring in 1.6–5.3% of the community-dwelling elderly [14, 15], may exacerbate the risk of falls. However, studies evaluated the falls experience in older adults with sarcopenia and knee OA separately [10–12, 16].

With this in mind, this cross-sectional study aimed to test the hypothesis that older adults with coexisting sarcopenia and knee OA had an increased experience of falls with the goal of suggesting a new concept “sarcopenic knee OA”, defined as concomitant of sarcopenia and knee OA, as a subgroup with higher risk of falls. This study would serve as a framework for future studies toward the establishment of fall prevention strategy.

## Methods

### Participants

This study is a part of an aging and OA project with the goal of establishing epidemiologic indexed towards development of effective preventive strategy for knee OA and extension of healthy life span. In this cross-sectional study, participants were identified through the medical record system and recruited from a community orthopedic clinic located in a rural mountainous community in Hiroshima and Kyoto, Japan. We recruited participants who were visiting the clinic for the conservative treatment of knee OA in January 2014 within a 3-day window. All recruited participants had a history of pain in one or both knees. The required sample size was not calculated and there was no limit to the maximum number of recruited participants. The eligibility criteria of the current cross-sectional study was as follows: (1) age ≥ 60 years and (2) ability to walk independently on a flat surface without any ambulatory assistive device. Two experienced physical therapists visually checked the participant’s walking ability on a flat surface. Since radiography was available only for painful knee, this study could not consider bilateral knee OA cases separately from unilateral cases. This study included participants with age ≥ 60 years because knee pain is common in community-dwelling older adults in Japan [17] and is recommended criteria for sarcopenia diagnosis according to Asian Working Group for Sarcopenia (AWGS) [18]. The exclusion criteria were (1) a history of knee surgery, (2) rheumatoid arthritis, (3) periarticular fracture, or (4) present neurological problems such as hemiplegia and Parkinson’s disease, all of which were assessed by medical record. There was no limit for time interval between these disease condition and study recruitment. This study was approved by the ethics committees in Kyoto University Graduate School and Faculty of Medicine and conducted in accordance with the principles of the Declaration of Helsinki. Written informed consent was obtained from all participants before their enrollment. This study was carried out in accordance with STROBE statement (see Supplemental Appendix S1).

### Measurements

Skeletal muscle mass index (SMI), handgrip strength (i.e., a metric of muscle strength), gait velocity (i.e., a metric of physical performance), and radiographic knee OA were evaluated. Fall experience was evaluated as outcome measurement. All outcome measures were evaluated by experienced physical therapists with > 6 years of clinical experience in musculoskeletal disorders and a postgraduate master’s degree qualification at the time of participant inclusion. Demographic characteristics and knee OA-related self-reported measures of knee pain and disability were also assessed as participant characteristics and/or covariates.

#### Assessment of sarcopenia

A bioelectrical impedance data acquisition system (Inbody 430; Biospace Co., Ltd., Seoul, Korea) was used to determine bioelectrical impedance in accordance with manufacture instruction [19]. This system uses electrical current at different frequencies (5, 50, and 250 kHz) to directly measure the amount of extracellular and intracellular water in the body and has been used in the previous epidemiological studies [20, 21]. The data acquisition system calculated the resistance value and muscle mass of the respective body parts (right arm, left arm, right leg, left leg, and trunk). The participants stood on 2 metallic electrodes and held metallic grip electrodes. The appendicular skeletal muscle mass was determined using the segmental body composition and muscle mass excluding the trunk part. SMI was calculated by dividing the skeletal muscle mass by height in square meters (kg/m^2^) [22]. The handgrip strength in both hands was measured using a hand dynamometer (Smedley Style Hand Grip Dynamometer; Tsutsumi Seisakusho Co., Ltd., Tokyo, Japan) [23] that shows excellent test-retest reliability [24]. The participants kept their arms by the sides of their body and squeezed the dynamometer using maximum isometric effort. Other body movements were prohibited [23]. To assess gait velocity, the participants were instructed to walk for 10 m at a self-selected speed. A trained examiner measured the time taken to walk 10 m with a stopwatch (TD-392; TANITA Corp., Tokyo, Japan), in accordance with a previously suggested method [25]. Gait velocity (m/s) was manually calculated as 10 m divided by the time needed to walk 10 m.

We defined sarcopenia as the presence of both low muscle function (low physical performance or low muscle strength) and low muscle mass in accordance with the recommended diagnostic algorithm of the AWGS [18]. If a participant had both low muscle function (slow walking speed [0.8 m/s] or low grip strength [< 26 and < 18 kg for men and women, respectively]) and low SMI (< 7.0 and < 5.7 kg/m^2^ for men and women, respectively), sarcopenia was diagnosed [18]. The prevalences of presarcopenia (i.e., low SMI without low muscle function) and severe sarcopenia (i.e., low SMI with slow walking speed and low grip strength) were provided for descriptive purposes.

#### Radiographic OA severity in knee joint

The radiographic severity of the tibiofemoral joint in index knee was assessed by a trained examiner (HI) using the original version of the Kellgren and Lawrence (K&L) grading system [26]. The index knee was defined as the more painful knee in the past or present. If a participant considered the pain in both knees to be equal, the index knee was randomly selected using a computer-generated permuted block randomization scheme [27]. We previously reported excellent intra-examiner (κ: 0.876; 95% confidence interval [CI]: 0.829, 0.924) and inter-examiner (κ: 0.845; 95% CI: 0.793, 0.897) reliability scores [28]. The presence of radiographic OA was defined as K&L grade ≥ 2.

#### Assessment of falls

A fall was defined as unintentionally coming to rest on the ground or at some other lower level, not because of a major intrinsic event (e.g., stroke) or an overwhelming external force (e.g., impact from a moving vehicle). Falls in the previous 12 months were evaluated using a self-reported question: “How many times did you have a fall within the past year?” An individual was considered a faller and recurrent faller if he or she has had at least 1 fall and 2 falls in the preceding 12 months, respectively. This 12-month falls recall questionnaire has been used in the previous epidemiological studies [5, 7, 8] and is acceptable alternative to prospective daily falls questionnaire in community-dwelling older adults [29].

#### Participant characteristics and covariates

Data on age, sex, and height were self-reported by the participants. Body mass was measured on a digital scale, with the participants dressed but not wearing shoes. Body mass index (BMI) was calculated by dividing the body mass (kg) by height in square meters (m^2^). The knee pain severity and self-reported disability were evaluated using the Japanese Knee Osteoarthritis Measure (JKOM) subcategories “pain and stiffness” (0–32 points) and “activities of daily living” (0–40 points) as a person-specific assessment [30]. The concurrent and construct validities of the JKOM were established by comparing with the Western Ontario and McMaster Universities Arthritis Index and the Medical Outcomes Study 36-item Short-form Health Survey [30].

### Classification based on sarcopenia and knee OA

**Fig. 1:**
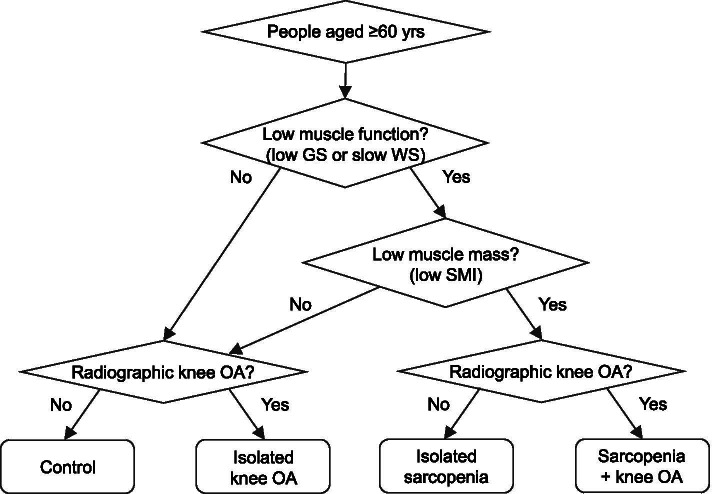
Diagnostic algorithms for the classification of four subgroups determined by sarcopenia and knee OA. People with sarcopenia + knee OA was identified as the coexistence of sarcopenia and radiographic knee OA (Kellgren and Lawrence grade ≥ 2). In the diagnosis of sarcopenia, we used the Asian Working Group criteria [18]. Specifically, sarcopenia was diagnosed if a participant had both low muscle function (slow walking speed [0.8 m/s] or low grip strength [< 26 kg and < 18 kg for men and women, respectively]) and low SMI (< 7.0 kg/m^2^ and < 5.7 kg/m^2^ for men and women, respectively). GS, grip strength; OA, osteoarthritis; SMI, skeletal muscle mass index; WS, walking speed; yrs., years

All subjects were allocated to one of the following four subgroups based on sarcopenia and radiographic OA in index knee: (1) control (non-knee OA and non-sarcopenia), (2) isolated sarcopenia, (3) isolated knee OA, and (4) sarcopenia + knee OA. Figure 1 shows diagnostic algorithms for the classification of four subgroups determined by sarcopenia and knee OA.

### Statistical analyses

We statistically analyzed the differences among the four groups using analysis of variance with a subsequent post hoc Tukey-Kramer test for parametric continuous variables, Kruskal-Wallis test with subsequent post hoc Steel-Dwass test for nonparametric continuous variables, and Fisher’s exact test for categorical variables. The normality of continuous variables was assessed using the Shapiro-Wilk test.

Binary logistic regression analyses were performed to assess the relationship between the four subgroups, an independent variable, and a fall (≥1 fall; 0: no, 1: yes) or recurrent falls (≥2 falls; 0: no, 1: yes) in the preceding 12 months, a dependent variable. In this analysis, the four subgroups were included as a dichotomous variable, such as control (reference), isolated sarcopenia (0: no; 1: yes), isolated sarcopenia (0: no; 1: yes), and sarcopenia + knee OA (0: no; 1: yes). As recurrent falls may have different risk factors and have been associated with increased physician contact and functional decline [5–8], this parameter (≥2 falls) was also included as a dependent variable in a separate binary logistic regression model. Ordinal logistic regression analysis was also performed, and falls were included as an ordinal dependent variable (1: no fall, 2: 1 fall, 3: ≥2 falls). In these analyses, age (continuous), female sex, and BMI (continuous) were included as covariates. These covariates were chosen a priori based on clinical judgment and their potential association with sarcopenia and knee OA [31, 32], not according to the causal pathway. The knee pain intensity (continuous) was further included as a covariate in a separate post-hoc logistic regression analysis.

To test the interaction between sarcopenia and radiographic knee OA, another logistic regression analysis was performed with sarcopenia (0: no; 1: yes), knee OA (0: no; 1: yes), and their interaction term (i.e., sarcopenia × knee OA) as independent variables. In this analysis, the dependent variable and covariates were included as mentioned above. Data analyses were performed using JMP 14.0 (SAS Institute, Cary, NC, USA).

## Results

Of the 302 evaluated participants, 11 (3.6%) were excluded because of missing data for JKOM score (*n* = 4), muscle mass (*n* = 5) and falls experience (*n* = 2). Thus, 291 participants (age: 60–90 years, 78.7% women) were finally included in the study (i.e., complete case analysis). Table 1 compares the participant characteristics among the control (*n* = 90; 70.3 ± 6.4 years), isolated sarcopenia (*n* = 12; 75.3 ± 7.3 years), isolated knee OA (*n* = 164; 72.9 ± 6.6 years), and sarcopenia + knee OA (*n* = 25; 78.7 ± 7.5 years) groups. Higher knee pain and disability, assessed by JKOM scores of pain and stiffness and activities of daily living, were prominent in participants with coexisting of sarcopenia and knee OA. Single and recurrent falls in the preceding 12 months were experienced by 60 (20.6%) and 21 (7.2%) participants, respectively. Eleven participants (age: 71.2 ± 8.5 years; BMI: 23.0 ± 2.2 kg/m^2^; 81.8% women) with missing data had similar demographic characteristics to 291 participants without missing data (age: 72.7 ± 7.0 years; BMI: 24.1 ± 3.7 kg/m^2^; 78.7% women).

**Table 1:** Participant characteristics (*n* = 291)

Variable	Mean ± SD or no. (%)				*p*-value
	Control n = 90	Isolated sarcopenia n = 12	Isolated knee OA n = 164	Sarcopenia + knee OA *n* = 25	
Age, years	70.3 ± 6.4	75.3 ± 7.3	72.9 ± 6.6^†1^	78.7 ± 7.5^†1,3^	< 0.001
Female	68 (75.6)	8 (66.7)	131 (79.9)	22 (88.0)	0.393
Height, cm	154.9 ± 7.4	152.1 ± 5.2	154.6 ± 7.1	149.8 ± 6.4^†1,3^	0.007
Mass, kg	56.7 ± 9.1	48.1 ± 8.2^†1^	59.4 ± 9.9^†2^	51.7 ± 9.7^†3^	< 0.001
BMI, kg/m^2^	23.6 ± 3.0	20.7 ± 2.8^†1^	24.8 ± 3.8^†1,2^	23.1 ± 4.5	< 0.001
Obesity^*1^	3 (3.3)	0 (0.0)	14 (8.5)	1 (4.0)	0.282
Index knee K&L grade					< 0.001
Grade 0	2 (2.2)	1 (8.3)	0 (0)	0 (0)	
Grade 1	88 (97.8)	11 (91.7)	0 (0)	0 (0)	
Grade 2	0 (0)	0 (0)	107 (65.3)	11 (44.0)	
Grade 3	0 (0)	0 (0)	37 (22.6)	11 (44.0)	
Grade 4	0 (0)	0 (0)	20 (12.2)	3 (12.0)	
JKOM, points					
Pain and stiffness^*2,3^	5.43 ± 5.09; 4.5 [1, 8]	6.08 ± 6.83; 4.5 [0, 12]	8.87 ± 6.49; 8 [4, 13]^†1^	8.20 ± 5.69; 8 [3, 12]	< 0.001
Activities of daily living^*2,3^	4.14 ± 4.87; 2 [1, 7]	5.75 ± 5.85; 4.5 [0, 11]	7.47 ± 6.97; 5 [2, 12]^†1^	9.68 ± 6.05; 9 [6, 14]^†1^	< 0.001
Physical function					
Gait velocity, m/s	1.27 ± 0.21	1.06 ± 0.26^†1^	1.18 ± 0.22^†1^	1.01 ± 0.24^†1,3^	< 0.001
Handgrip strength, kg	25.8 ± 6.7	17.1 ± 5.3^†1^	25.5 ± 6.4^†2^	15.9 ± 3.8^†1,3^	< 0.001
Muscle volume					
Skeletal muscle index, kg/m^2^	6.25 ± 0.80	5.50 ± 0.91^†1^	6.47 ± 0.78^†2^	5.41 ± 0.44^†1,3^	< 0.001
Pre-sarcopenia	37 (41.1)	0 (0.0)	37 (22.6)	0 (0.0)	NA
Sarcopenia	0 (0.0)	12 (100.0)	0 (0.0)	25 (100.0)	NA
Severe sarcopenia	0 (0.0)	11 (91.7)	0 (0.0)	24 (96.0)	NA

BMI, body mass index; JKOM, Japanese Knee Osteoarthritis Measure; K&L grade, Kellgren and Lawrence grade; NA, not applicable; OA, osteoarthritis; SD, standard deviation

*p*-values are based on unadjusted analyses (analysis of variance, Kruskal-Wallis test, or Fisher’s exact test)

^*1^ Obesity was defined as BMI ≥30 kg/m^2^

^*2^ Higher scores indicate severe knee pain or severe disability

^*3^ Median [interquartile range] was also provided because of the scattered distribution of the answered items

^†1^ p < 0.05 compared to control ^†2^ p < 0.05 compared to isolated sarcopenia

^†3^ *p* < 0.05 compared to isolated knee OA

**Fig. 2:**
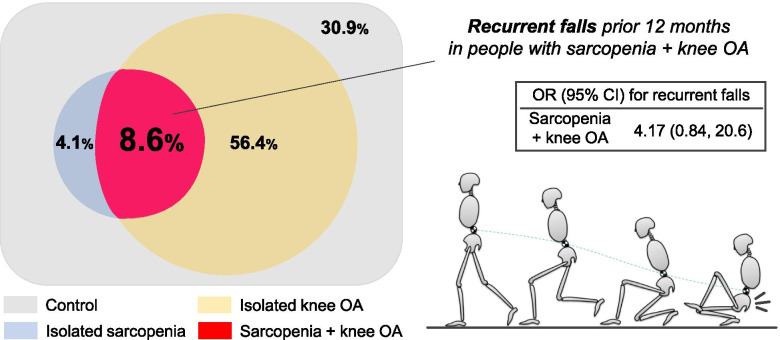
Graphical abstract. Of 291 participants, 25 (8.6%) had coexisting sarcopenia and knee osteoarthritis (OA). People with coexisting sarcopenia and knee OA demonstrated higher prevalence of recurrent falls in the preceding 12 months (odds ratio: 4.17; 95% confidence interval: 0.84, 20.6) after adjustment for age, sex, and body mass index

Table 2 compares the incidence of single and multiple falls among the four subgroups. Participants with isolated sarcopenia more frequently (41.7%) had a single fall than those in the other 3 groups, and those with sarcopenia + knee OA more frequently (16.0%) had multiple falls than participants in the other 3 groups. Binary logistic regression analysis revealed that participants with isolated sarcopenia displayed higher prevalence of single fall experience than the control group after adjustment for age, sex, and BMI (odds ratio [OR]: 4.63; 95% CI: 1.28, 16.7; *p* = 0.019). Notably, participants with sarcopenia + knee OA displayed higher prevalence of both single (OR: 2.53; 95% CI: 0.91, 7.04; *p* = 0.075) and recurrent fall experience (OR: 4.17; 95% CI: 0.84, 20.6; *p* = 0.088) than the control group after adjustment for age, sex, and BMI. Ordinal logistic regression analysis also showed that participants with isolated sarcopenia (proportional OR: 4.09; 95% CI: 1.18, 14.2; *p* = 0.027) and sarcopenia + knee OA (proportional OR: 2.84; 95% CI: 1.05, 7.70; *p* = 0.040) had an increased probability of experiencing single or multiple falls. Additional inclusion of knee pain intensity as a covariate showed similar results; however, the relationship between sarcopenia + knee OA and recurrent falls was attenuated (OR: 3.75; 95% CI: 0.75, 18.7; *p* = 0.114). The sarcopenia × knee OA interaction in binary logistic regression analysis was not statistically significant in the association with a single fall (*p* = 0.207) and recurrent falls (*p* = 0.645). Figure 2 shows the graphical abstract.

**Table 2:** Results of binary and ordinal logistic regression analyses showing the relationship between the four subgroups and fall experience in older adults

Variable	No. (%) of participants			OR (95% CI)†		Proportional OR (95% CI)†
	No fall	1 fall	2 ≤ falls	1 ≤ fall	2 ≤ falls	
Control (n = 90)	72 (80.0)	13 (14.4)	5 (5.6)	–	–	
Isolated sarcopenia (n = 12)	6 (50.0)	5 (41.7)	1 (8.3)	**4.63 (1.28, 16.7)**	2.19 (0.21, 22.6)	**4.09 (1.18, 14.2)**
Isolated knee OA (n = 164)	117 (71.3)	36 (22.0)	11 (6.7)	1.50 (0.79, 2.84)	1.03 (0.33, 3.24)	1.46 (0.77, 2.74)
Sarcopenia + knee OA (n = 25)	15 (60.0)	6 (24.0)	4 (16.0)	2.53 (0.91, 7.04)	4.17 (0.84, 20.6)	**2.84 (1.05, 7.70)**

95% CI, 95% confidence interval; OR, odds ratio

Bold type indicates a statistically significant result

† OR (95% CI) and proportional OR (95% CI) values of the four subgroups (control: reference) were calculated to indicate their predictive ability for fall experience (1 ≤ fall or 2 ≤ falls) while simultaneously including (1-step model) age, female sex, and body mass index. In the analysis for any falls experience (1 ≤ fall), recurrent falls (2 ≤ falls) were also included in a dependent variable

## Discussion

The objective of this work was to test the hypothesis that older adults with coexisting sarcopenia and knee OA had an increased risk of falls. Of 291 orthopedic clinic participants, we found that 8.6% participants had both sarcopenia and knee OA. This prevalence rate is in line with previous reports, occurring in 1.6–5.3% of the community-dwelling elderly [14, 15]. Most notably, these participants had a higher prevalence of recurrent falls in the preceding 12 months than those without sarcopenia and knee OA, which was not clearly confirmed in participants with sarcopenia or knee OA alone.

Sarcopenia and OA have been postulated to be coexisting conditions [33]. However, falls in these patients have been often investigated separately [10–12, 16], thereby precluding a holistic understanding of this common coexisting condition. Here, we found participants with coexisting of sarcopenia and knee OA displayed increased recurrent falls experience, suggesting that concomitant of sarcopenia and knee OA may contribute to the incidence of falls, or vice versa. It should be noted that this study included orthopedic clinic patients who had a history of pain in one or both knees, which is also an important confounder [34]. Including knee pain intensity into the logistic regression model showed similar results but slightly weakened the observed relationship, indicating that the increased recurrent falls experience in people with sarcopenia and knee OA may be partly attributed to severe knee pain. Conversely, the contribution of knee pain on the recurrent falls experience also indicates that recurrent falls experience in participants with coexisting of sarcopenia and knee OA cannot be fully explained by physical frailty, characterized by diminished strength, endurance, and reduced physiologic function that increases an individual’s vulnerability.

Several works have been done on sarcopenia and knee OA as an independent risk factor for falls in older adults [13, 16]. The current study expands on the previous knowledge by showing that concomitant of these conditions may further increase the risk of falls. Although underlying mechanism in the increased falls experience in participants with coexisting sarcopenia and knee OA is not fully understood, severe knee pain in those participants may partially explain the increased fall experience. Chronic pain interferes with the necessary cognitive activity to prevent a fall [35]. Successful avoidance or interruption of falls typically requires a cognitively mediated physical maneuver, such as a quick reaction during ambulation. Indeed, the ability of people with knee OA to avoid obstacles is impaired [36], and pain relief partially restores this ability [37]. Further studies addressing the role of pain for increased recurrent falls in people with coexisting of sarcopenia and knee OA would be desirable.

Despite the interest in the topic, limited studies have investigated the clinical manifestations of people with coexisting of sarcopenia and knee OA, “sarcopenic OA” [15, 38]. Chung et al. revealed that people with coexisting sarcopenia and knee OA showed increased risks of metabolic syndrome and insulin resistance [15], which may further aggravate these two chronic conditions. Another study, reported by Ho et al., revealed that sarcopenia in patients with end-stage knee OA is common, but these patients displayed a similar clinical and functional improvement compared to those without sarcopenia after total knee arthroplasty at 12-month after the surgery [38]. The current study could prompt future studies on characterization of the “sarcopenic OA” and how to effectively manage this common and potentially debilitating condition.

Although this study provides a new perspective to the clinical impacts of the concomitant of sarcopenia and knee OA, it has some limitations. First, the cross-sectional design with small sample size limits our analysis. Concomitant of sarcopenia and knee OA may be a consequence of previous falls. Since this study did not calculate required sample size, our findings are hypothesis-generating, which should be validated in future large cohort studies. Second, the lack of information on the pain profile in other joints and drug use including pain medication is also an important limitation. Since all participants, including controls, had a history of knee pain, pain medication may influence the relationship between sarcopenia, knee OA, and falls experience. In addition, we did not evaluate cognitive impairment which may cause recall bias for falls assessment. Although we minimized participants’ fatigue and assessment time, future studies should consider these risk factors for recurrent falls [39, 40]. Also, this study did not consider the sarcopenia stage in statistical analysis and most of the participants with sarcopenia displayed severe sarcopenia, which may influence the generalizability of our results. Finally, this study did not clarify the cause of falls; thus, the mechanism behind the higher prevalence of recurrent falls in people with coexisting of sarcopenia and knee OA is still unclear. We cannot discount the possibility that falls experience included uncontrolled falls with physical and emotional disturbances.

This study has several strengths: (1) we recruited orthopedic clinic patients who had a history of knee pain, which would help clinician to identify potentially high risk subgroup for recurrent falls and subsequent appropriate management for their patients; and (2) experienced physical therapists evaluated all outcomes to ensure validated and reliable measurements.

Taken together, this study suggests a new concept of knee OA, “sarcopenic knee OA”, as a subgroup associated with higher risk of falls. We found that people with coexistence of sarcopenia and knee OA have an increased likelihood of experiencing recurrent falls in the preceding 12 months. Although small sample size limits making a strong conclusion, a thorough investigation of sarcopenia-OA interaction may provide novel insights into the pathomechanics of recurrent falls in older adults.

## Supplementary Information

**Additional file 1.** STROBE Statement.

## Data Availability

The datasets used and/or analyzed during the current study are available from the corresponding author on reasonable request.

## Abbreviations

BMI: Body mass index
JKOM: Japanese Knee Osteoarthritis Measure
K&L grade: Kellgren and Lawrence grade
OA: Osteoarthritis
SMI: Skeletal muscle mass index
