# Supplementary material for: Increased recurrent falls experience in older adults with coexisting of sarcopenia and knee osteoarthritis: a cross-sectional study
Source: BMC Geriatr. 2021 Dec 15;21:698. doi: 10.1186/s12877-021-02654-4 (PMC8672583; doi:10.1186/s12877-021-02654-4)
Supplement: Supplementary file 1 — Additional file 1. STROBE Statement. [file 12877_2021_2654_MOESM1_ESM.doc]

STROBE Statement

|  | Item No | Recommendation |
| --- | --- | --- |
| **Title and abstract** | 1 | (*a*) Indicate the study’s design with a commonly used term in the title or the abstract **[ 1 ]** |
|  |  | (*b*) Provide in the abstract an informative and balanced summary of what was done and what was found **[ 1 ]** |
| Introduction | | |
| Background/rationale | 2 | Explain the scientific background and rationale for the investigation being reported **[ 3 ]** |
| Objectives | 3 | State specific objectives, including any prespecified hypotheses **[ 3 ]** |
| Methods | | |
| Study design | 4 | Present key elements of study design early in the paper **[ 4 ]** |
| Setting | 5 | Describe the setting, locations, and relevant dates, including periods of recruitment, exposure, follow-up, and data collection **[ 4, 5 ]** |
| Participants | 6 | (*a*) *Cohort study*—Give the eligibility criteria, and the sources and methods of selection of participants. Describe methods of follow-up **[ N/A ]**  *Case-control study*—Give the eligibility criteria, and the sources and methods of case ascertainment and control selection. Give the rationale for the choice of cases and controls **[ N/A ]**  *Cross-sectional study*—Give the eligibility criteria, and the sources and methods of selection of participants **[ 4, 5 ]** |
|  |  | (*b*) *Cohort study*—For matched studies, give matching criteria and number of exposed and unexposed **[ N/A ]**  *Case-control study*—For matched studies, give matching criteria and the number of controls per case **[ N/A ]** |
| Variables | 7 | Clearly define all outcomes, exposures, predictors, potential confounders, and effect modifiers. Give diagnostic criteria, if applicable **[ 5 ]** |
| Data sources/ measurement | 8* | For each variable of interest, give sources of data and details of methods of assessment (measurement). Describe comparability of assessment methods if there is more than one group **[ 5-8 ]** |
| Bias | 9 | Describe any efforts to address potential sources of bias **[ 5-8 ]** |
| Study size | 10 | Explain how the study size was arrived at **[ N/A ]** |
| Quantitative variables | 11 | Explain how quantitative variables were handled in the analyses. If applicable, describe which groupings were chosen and why **[ 8, 9 ]** |
| Statistical methods | 12 | (*a*) Describe all statistical methods, including those used to control for confounding **[ 8, 9 ]** |
|  |  | (*b*) Describe any methods used to examine subgroups and interactions  **[ 8, 9 ]** |
|  |  | (*c*) Explain how missing data were addressed **[ 8, 9 ]** |
|  |  | (*d*) *Cohort study*—If applicable, explain how loss to follow-up was addressed **[ N/A ]**  *Case-control study*—If applicable, explain how matching of cases and controls was addressed **[ N/A ]**  *Cross-sectional study*—If applicable, describe analytical methods taking account of sampling strategy **[ N/A ]** |
|  |  | (*e*) Describe any sensitivity analyses **[ N/A ]** |

Continued on next page

| Results | | |
| --- | --- | --- |
| Participants | 13* | (a) Report numbers of individuals at each stage of study—eg numbers potentially eligible, examined for eligibility, confirmed eligible, included in the study, completing follow-up, and analysed **[ 9 ]** |
|  |  | (b) Give reasons for non-participation at each stage **[ 9 ]** |
|  |  | (c) Consider use of a flow diagram **[ N/A ]** |
| Descriptive data | 14* | (a) Give characteristics of study participants (eg demographic, clinical, social) and information on exposures and potential confounders **[ 10 ]** |
|  |  | (b) Indicate number of participants with missing data for each variable of interest **[ 9 ]** |
|  |  | (c) *Cohort study*—Summarise follow-up time (eg, average and total amount) **[ N/A ]** |
| Outcome data | 15* | *Cohort study*—Report numbers of outcome events or summary measures over time **[ N/A ]** |
|  |  | *Case-control study—*Report numbers in each exposure category, or summary measures of exposure **[ N/A ]** |
|  |  | *Cross-sectional study—*Report numbers of outcome events or summary measures **[ N/A ]** |
| Main results | 16 | (*a*) Give unadjusted estimates and, if applicable, confounder-adjusted estimates and their precision (eg, 95% confidence interval). Make clear which confounders were adjusted for and why they were included **[ 10 ]** |
|  |  | (*b*) Report category boundaries when continuous variables were categorized **[ N/A ]** |
|  |  | (*c*) If relevant, consider translating estimates of relative risk into absolute risk for a meaningful time period **[ N/A ]** |
| Other analyses | 17 | Report other analyses done—eg analyses of subgroups and interactions, and sensitivity analyses **[ N/A ]** |
| Discussion | | |
| Key results | 18 | Summarise key results with reference to study objectives **[ 11 ]** |
| Limitations | 19 | Discuss limitations of the study, taking into account sources of potential bias or imprecision. Discuss both direction and magnitude of any potential bias **[ 13 ]** |
| Interpretation | 20 | Give a cautious overall interpretation of results considering objectives, limitations, multiplicity of analyses, results from similar studies, and other relevant evidence **[ 11, 12, 13, 14 ]** |
| Generalisability | 21 | Discuss the generalisability (external validity) of the study results **[13]** |
| Other information | | |
| Funding | 22 | Give the source of funding and the role of the funders for the present study and, if applicable, for the original study on which the present article is based **[ 15 ]** |
